# Costal exostoses as an unusual cause of spontaneous hemothorax

**DOI:** 10.1186/s13019-015-0342-6

**Published:** 2015-10-27

**Authors:** Jeong Seob Yoon, Jong Bum Kwon, Chan Beom Park, Jong Hui Suh

**Affiliations:** 1 Department of Thoracic and Cardiovascular Surgery, Incheon St. Mary’s Hospital, The Catholic University of Korea, 56, Dongsu-Ro, Bupyeong-Gu, Incheon, 403-720 Republic of Korea; 2 Department of Thoracic and Cardiovascular Surgery, Daejeon St. Mary’s Hospital, College of Medicine, The Catholic University of Korea, 64, Daeheong-Ro, Jung-Gu, Daejeon, 34943 Republic of Korea

**Keywords:** Costal exostoses, Diaphragmatic injury, Hemothorax

## Abstract

A 20-year-old male presented with chest pain lasting several days. A radiologic examination revealed pleural effusion in the right hemithorax. Video-assisted thoracoscopic surgery demonstrated a bleeding focus at the diaphragm caused by injury due to a costal exostosis.

## Background

Injury to the diaphragm is usually caused by blunt or penetrating trauma. Although gunshot and stab wounds are main causes of penetrating diaphragm injury, abnormalities of the bony thorax, such as costal exostoses, may also result in the diaphragm injury. We report heein an unusual case of spontaneous hemothorax associated with diaphragmatic laceration due to costal exostosis.

## Case presentation

A 20-year-old man without a history of trauma was admitted to an emergency department with right-sided pleuritic chest pain. He had a history of surgical resection of a bony spur on the shoulder, and a diagnosis of osteochondroma on both radii 7 and 2 years prior, respectively.

**Fig. 1:**
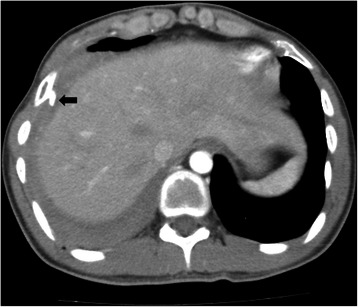
Chest computed tomography scan showing pleural effusion In the right hemithorax. The black arrow indicates a protruding rib spiculation

A pleural effusion was noted in the right hemithorax, and chest computed tomography revealed a focal bony protrusion in the anterior arc of the right sixth and left fourth ribs (Fig. 1). A diagnostic pleural tap revealed dark blood in the thoracic cavity, and so video-assisted thoracoscopic exploration was conducted to identify the focus of the intrathoracic bleeding and treatment of a spontaneous hemothorax.

**Fig. 2:**
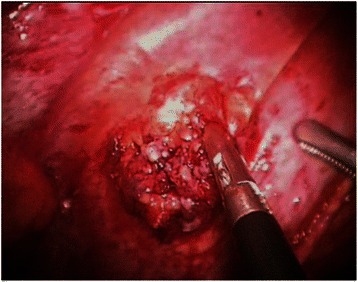
Operating field showing the irregular diaphragmatic surface resulting from repetitive injury due to a costal exostosis

A 1.5 cm laceration was observed on the diaphragmatic surface that was covered with a blood clot (Fig. 2), and an inward-facing bony spiculation from the anterior arc of the right sixth rib was revealed. The costal exostosis was resected and eroded diaphragm was repaired with an Endo-GIA stapler (Ethicon Endo-Surgery, Cincinnati, OH, USA). The patient’s postoperative course was uneventful. The chest tube was removed on the third postoperative day and the patient was discharged on the seventh postoperative day without complication. The bony spur of left fourth rib was not resected, and the patient was followed without recurrent hemothorax for 4 years.

## Discussion

We present a case of a young male patient with spontaneous hemothorax, the cause of which was demonstrated by video-assisted thoracoscopy to be bleeding from a diaphragmatic injury incurred by a costal exostosis.

Costal exostosis is an abnormal inward protrusion of osteochondoma arising from costal cartilage.

Osteochondroma is a common benign neoplasm of the rib [1]. Osteochondromas rarely present with a pattern of multiple exostoses, which are usually autosomal dominant in nature. These hereditary multiple exostoses are benign clinical conditions and are usually well tolerated, with patients achieving an average lifespan. Most osteochondromas are usually nontender, painless, and slow-growing masses. However, osteochondromas occurring in adolescence (after puberty) or in adult patients can grow in size and become symptomatic as a result of mechanical irritation of the surrounding soft tissues or peripheral nerves, spinal cord compression, or vascular injury.

The pathogenesis of costal exostoses is unclear, but it appears that developmental growth defect of the fibrous tissue (perichondrium) covering the epiphyseal plate may result in lateral growth of the epiphyseal cartilage plate instead of the normal downward growth toward the metaphysis. This abnormal growth leads to an inward protrusion of the rib cartilage [2]. The mechanisms underlying diaphragmatic injury due to exostosis include direct perforation by a sharp bony spur or repetitive erosion by particularly pointed bony extrusions. Injury to the diaphragm, pleura, heart, and lung have all been reported [3–6], and can cause a life- threatening condition, as in our case.

Surgical removal of osteochondromas is not usually indicated, especially in childhood. However, surgical resection is indicated for osteochondromas developing in adolescence after puberty or in adult patients with pain, increased size, and mechanical complications. Simansky et al. [3] reviewed eight cases of intrathoracic exostoses and found that in five patients the bleeding originated from the erosion of the parietal pleura, and in three cases of hemothorax developed as a result of irritation of the diaphragm. Two cases were treated using a video-assisted thoracoscopic procesure, three cases were managed by thoracotomy, and three cases were treated by drainage only.

## Conclusion

While multiple rib exostoses occur only rarely, hereditary multiple exostosis should be considered in cases of young patients with nontraumatic hemothorax, and prophylactic surgical removal of intrathoracic exostosis should be considered even in asympatomatic patients with the presentation of an inward bony spiculation.
